# Magnetic resonance imaging findings of the primitive neuroectodermal tumour in lumbosacral spinal cord in a cat

**DOI:** 10.1002/vms3.1283

**Published:** 2023-09-23

**Authors:** Takamasa Itoi, Kenji Kutara, Ikki Mitsui, Natsuki Akashi, Teppei Kanda, Keisuke Sugimoto, Yuki Shimizu, Kazuaki Yamazoe

**Affiliations:** ^1^ Faculty of Veterinary Medicine Okayama University of Science Imabari Ehime Japan

**Keywords:** cat, lumbosacral, magnetic resonance imaging, primitive neuroectodermal tumour

## Abstract

A 5‐year‐old, castrated, male domestic short‐haired cat presented with neurological deficits in the pelvic limbs, back pain and dysuria. Magnetic resonance imaging showed a mass lesion caudal to the L4 vertebrae. In addition, suspected haemorrhage was observed at the cranial aspect of the mass. There was no evidence to support the presence of extravertebral intrusion or vertebral body, osteolysis. Dorsal laminectomy and durotomy were performed to debulk the intraspinal mass. Histopathological and immunohistochemical assessment revealed a primitive neuroectodermal tumour (PNET). To our knowledge, this is the first report to describe the clinical and pathological features and imaging diagnosis of intraspinal PNET without extraspinal invasion in a cat.

## Graphical abstract

Graphical Abstract: A 5‐year‐old, castrated, male domestic short‐haired cat presented with neurological deficits in the pelvic limbs, back pain and dysuria. Magnetic resonance imaging showed a mass lesion caudal to the L4 vertebrae. In addition, suspected haemorrhage was observed at the cranial aspect of the mass. There was no evidence to support the presence of extravertebral intrusion or vertebral body, osteolysis. Dorsal laminectomy and durotomy were performed to debulk the intraspinal mass. Histopathological and immunohistochemical assessment revealed a primitive neuroectodermal tumour (PNET).

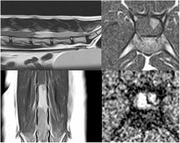

## 1 INTRODUCTION

Primitive neuroectodermal tumours (PNETs) are rare nervous system tumours. They occur most often in the head and very rarely in the spinal cord (Ma et al., 2020). In veterinary medicine, PNETs associated with the spinal cord have only been reported in canine, camelid and bovine patients. It has been reported that the canine (Hespel et al., 2021) and camelid (Weiss & Walz, 2009) spinal cord can be invaded by an extrapleural mass. Berrocal et al. (2005) reported a bovine intramedural tumour: Their report discussed only the pathological findings and did not include any imaging findings. In this report, we describe the clinical, imaging and pathological features of a cat with an intraspinal PNET.

## 2 CASE HISTORY

A 5‐year‐old, castrated, male, domestic, short‐haired cat was referred to neurological deficits in the pelvic limbs, back pain and dysuria. Lameness of the pelvic limbs had been reported for 2 months prior to referral and paralysis of the pelvic limbs for 10 days prior to referral. Neurological examination revealed severe ambulatory paraparesis without conscious proprioception in both pelvic limbs, whereas segmental spinal reflexes showed lower motor neuron signs (patella, cranial tibialis and gastrocnemius reflexes were near absent). Perineal reflex and deep pain were normal; however, dysuria was demonstrated. The remainder of the clinical examination was unremarkable. A complete blood cell count, serum biochemical evaluation, echocardiography and thoracic and abdominal radiographs identified no clinically significant abnormalities.

A 24‐G over‐the‐needle catheter was placed in the cephalic vein of the cat. The cat was premedicated with intravenous butorphanol tartrate (0.2 mg/kg) and intubated after induction with intravenous alfaxalone (5 mg/kg). General anaesthesia was maintained by mechanical ventilation with sevoflurane (2.5%–3%) and oxygen (2 L/min). Computed tomography (CT) imaging of spine (including the thorax and abdomen) was performed using a 16‐slice multi‐slice CT scanner (Aquilion Lightning, Canon Medical Systems). This identified no significant findings. In particular, there was no evidence to support tumour formation, metastasis or embolization of the aorta and no evidence of osteolysis.

Under the same anaesthetic condition, magnetic resonance imaging (MRI) was performed using a 1.5‐T superconducting unit (Vantage Elan; Canon Medical Systems) in the following planes: T1‐weighted imaging (T1WI) and T2‐weighted imaging (T2WI) in the sagittal, transverse and dorsal planes and T2‐star‐weighted imaging (T2*WI) in the transverse plane. T1WI in the sagittal, transverse and dorsal planes was performed after intravenous administration of 0.2 mg/kg bodyweight gadodiamide (Omniscan; Daiichi‐Sankyo Company).

**FIGURE 1:**
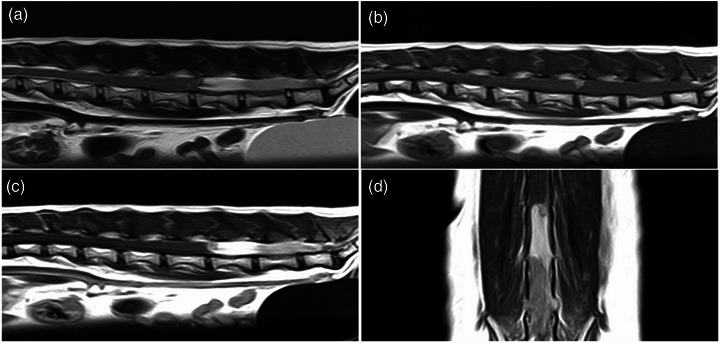
(A) T2‐weighted imaging (T2WI), (B) T1‐weighted imaging (T1WI), (C) contrast‐enhanced T1WI in the sagittal plane and (D) contrast‐enhanced T1WI in the horizontal plane. T2WI shows a hyperintense lesion at the level of the L5 vertebrae and a mild hyperintense lesion at the levels caudal to the L5 vertebrae. These lesions appeared isointense on T1WI. On contrast‐enhanced T1WI, the lesion showing hyperintensity on T2WI showed uniform contrast enhancement, whereas the lesion showing mild hyperintensity showed uniform mild contrast enhancement. On the cranial side of these findings, the lesion showed hyperintensity on T1WI.

**FIGURE 2:**
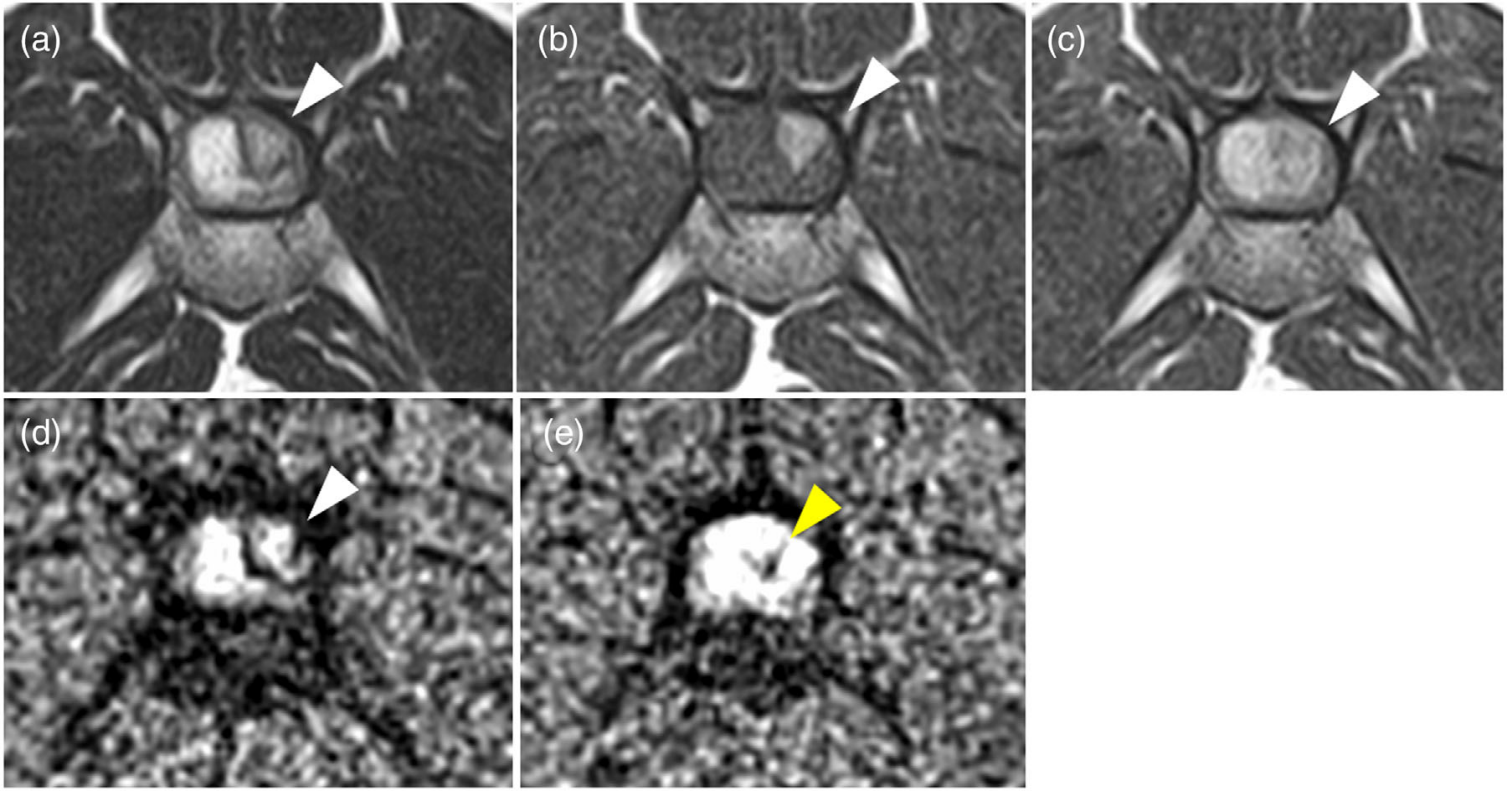
(A) T2‐weighted imaging (T2WI), (B) T1‐weighted imaging (T1WI), (C) contrast‐enhanced T1WI and (D) T2‐star‐weighted imaging (T2*WI) MR images in the transverse plane on the cranial side of the mass finding (at the level of the L4–5 intervertebral disc space). The findings indicate mild hyperintensity in T2WI, hyperintensity in T1WI and hypointensity at the margin on T2*WI appeared on the right dorsal side of the spinal cord (white arrowhead). In (E) T2*WI image of the level of cranial aspect of L5, a hypointense area was observed in the spinal cord (yellow arrowhead).

T2WI showed hyperintense spinal cord lesions at the level of the L5 vertebrae and mild hyperintense lesions caudal to the L5 vertebrae (Figure 1A). These lesions were isointense on T1WI (Figure 1B). On contrast‐enhanced T1WI, the lesion with hyperintensity on T2WI showed homogeneously contrast enhancement, whereas lesions with mild hyperintensity showed uniform mild contrast enhancement (Figure 1C,D). The imaging examinations did not indicate any extraspinal intrusion. Based on these findings, a spinal mass was suspected. There were no signal abnormalities cranial to L4. However, the spinal fluid lines around the spinal cord at the level of the L3–4 vertebrae were indistinct. Mild hyperintensity on T2WI, hyperintensity on T1WI and hypointensity at the margin on T2*WI were observed at the right dorsal aspect of the spinal cord at the level of the L4–5 intervertebral disc space (Figure 2A–D). In T2*WI image of the level of cranial aspect of L5, a hypointense area was observed in the spinal cord (Figure 2E), indicating haemorrhage. Cerebrospinal fluid collection from the lumbar spine (L5–6) was attempted but was unsuccessful. Based on the imaging appearance, a diagnosis of a spinal tumour was made. However, it was difficult to determine whether the tumour was intradural‐extramedullary or intramedullary.

Dorsal laminectomy and durotomy of the L4–7 vertebrae were performed 1 week after the initial investigations to improve pelvic limb paraparesis and back pain. The dura in the surgical field (L4–7) was dark‐pink, and caudal to L4 vertebral, it was partially torn with a honeycomb appearance. Durotomy was performed, and a dark red jelly‐like tumour was found occupying the space between the spinal nerve fibres. This was associated with multiple adjacent haematomas. The tumour was removed carefully to avoid damaging the spinal cord; however, the spinal cord parenchyma was partially dissolved the level of the L4 vertebra, suggesting a transition to myelomalacia. The patient initially recovered uneventually following surgery but experienced respiratory failure 12 h postoperatively. Mechanical ventilation was initiated, and the patient was treated with antibiotics (cefazolin sodium, 20 mg/kg, IV) and steroids (prednisolone, 1 mg/kg, SC); however, 2 days postoperatively, the patient died due to cardiac arrest. The cause of death was suspected to be the progression of myelomalacia. An autopsy was offered but declined.

**FIGURE 3:**
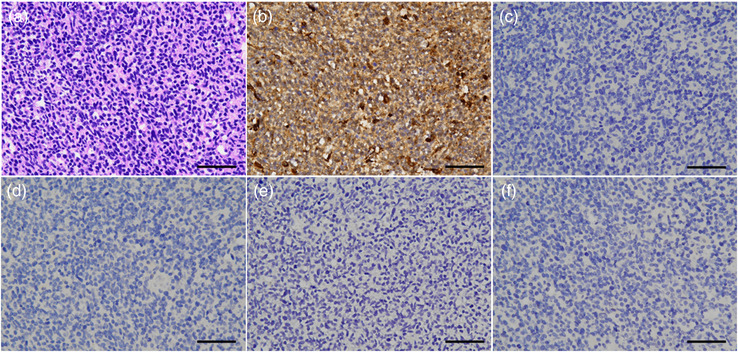
Histological and immunohistochemical features of the spinal mass. (A) ×400: The tumour is composed of dense sheets or short interlacing bundles of small round‐to‐short spindle cells. Haematoxylin and eosin staining; bar = 50 μm. (B) ×400: Tumour cells show cytoplasmic immunolabelling with vimentin; bar = 50 μm. (C) ×400: Tumour cells do not show immunolabelling with synaptophysin; bar = 50 μm. (D) ×400: Tumour cells do not show immunolabelling with glial fibrillary acidic protein (GFAP); bar = 50 μm. (E) ×400: tumour cells do not show immunolabelling with neurofilament protein; bar = 50 μm. (F) ×400: Tumour cells do not show immunolabelling with Olig‐2; bar = 50 μm.

The resected spinal mass was fixed in 10% neutral‐buffered formalin for 2 days, routinely processed and embedded in paraffin. Four‐micrometer‐thick sections were stained with haematoxylin and eosin for histopathological examination. The lesion was composed of dense sheets and short interlacing bundles of small, round‐to‐short spindle cells (Figure 3A). The cells had indistinct cell boundaries, scant pale eosinophilic cytoplasm and pleomorphic (round, oval or carrot‐shaped) hyperchromatic nuclei (Figure 3A) with an inconspicuous nucleolus and minimal anisocytosis and anisokaryosis. Three mitoses/10 high‐power fields (2.37 mm^2^) were observed using an ocular lens of field number 22, and a presumptive diagnosis of PNET was made. Immunohistochemical examination was performed to further characterize the neoplastic cells using the 12 antibodies listed in Table 1. The neoplastic cells showed positive staining only with the anti‐vimentin antibody (Figure 3B–F) but as immunoreactivity of the anti‐calretinin and CD45 antibodies had not been validated, the significance of these negative results was uncertain. A final diagnosis of PNET was made on the basis of histopathological and immunohistochemical results.

**TABLE 1:** Primary antibodies used for immunohistochemistry.

Antibody to	Host	Type, clone	Dilution	Source	Catalogue number
Human cytokeratin	Mouse	Monoclonal, AE1/AE3	RTU	Leica	PA0094
Porcine vimentin	Mouse	Monoclonal, V9	1:600	DAKO	M0725
Human CD3	Mouse	Monoclonal, LN10	RTU	Leica	PA0553
Human CD20	Rabbit	Polyclonal	1:200	Invitrogen	PA5‐16701
Human CD45	Mouse	Monoclonal, 2B11 + PD7/26	1:300	DAKO	M0701
Human Melan A	Mouse	Monoclonal, A103	1:75	DAKO	M7196
Human synaptophysin	Mouse	Monoclonal, 27G12	1:200	Leica	NCL‐L‐SYNAP‐299
Human neurofilament protein	Mouse	Monoclonal, 2F11	1:100	DAKO	M0762
Human calretinin	Mouse	Monoclonal, CAL6	1:200	Leica	NCL‐L‐CALRET‐566
Bovine GFAP	Rabbit	Polyclonal	RTU	DAKO	IS524
Recombinant Murine Olig‐2	Rabbit	Polyclonal	1:200	Millipore	AB9610
Bovine S100 A & B	Rabbit	Polyclonal	1:1600	DAKO	Z0311

Abbreviations: GFAP, glial fibrillary acidic protein; RTU, ready to use.

## 3 DISCUSSION

PNETs are rare malignant neuroendocrine tumours composed of small, undifferentiated or neuroectodermal cells (Ma et al., 2020). PNET is a member of the Ewing sarcoma family of tumours (Pu et al., 2021). In humans, the common sites of PNETs are the paraspinal and retroperitoneal areas and extremities (Ottóffy & Komáromy, 2020). Intraspinal PNET is rarer than intracranial PNET (Ma et al., 2020). These tumours have been reported only rarely in veterinary medicine. Central PNETs arising from the brain parenchyma represent 2.8% of all canine cases of primary intracranial neoplasia (Headley et al., 2009; Snyder et al., 2006). PNETs associated with the spinal cord have only been reported in canine (Hespel et al., 2021), camelid (Weiss & Walz, 2009) and bovine (Berrocal et al., 2005) patients. In the canine patient, an extrapleural mass invaded the spinal cord (Hespel et al., 2021), and the camelid patient had a vertebral tumour compressing the spinal cord (Weiss & Walz, 2009). As in the present case, an intradural tumour was described in the bovine patient; however, the report described only pathological findings and did not include any imaging findings (Berrocal et al., 2005). To our knowledge, this is the first report of a PNET occurring exclusively in the intraspinal and intradural regions in a cat.

In this case, the spinal tumour was isointense on T1WI, hyperintense on T2WI, and showed homogeneously contrast enhancement. Additionally, a suspected haemorrhage/haematoma was noted at the cranial aspect of the tumour. In humans, PNET has been reported to be hypointense or isointense on T1WI and hyperintense on T2WI with contrast enhancement (Cui & Zhao, 2022; Mechri et al., 2018; Nutman et al., 2007; Wang & Guo, 2017) and may be accompanied by MRI findings indicating intratumoural haemorrhage (Karthigeyan et al., 2021). The present findings were consistent with these reports. Additionally, isointensity on T1WI, hyperintensity on T2WI and homogeneous enhancement have been reported for other common intradural tumours, such as schwannomas, meningiomas, neurofibromas, lymphomas and metastasis (Cui & Zhao, 2022; Nutman et al., 2007). However, haemorrhage within spinal intradural tumours is rare. Spinal intradural Ewing's sarcomas may show a tendency to bleed (Karthigeyan et al., 2021), and spinal ependymomas can occasionally present with haemorrhagic manifestations (Terao et al., 2016). In veterinary medicine, intramedullary haemangiosarcomas in dogs can show hypointensity lesions in T2*WI, indicating haemorrhage (Hammond & Hecht, 2015; Mallol et al., 2021). However, they showed a mixed‐signal intensity in T2WI and T1WI, with ring‐like heterogeneous contrast enhancement (Mallol et al., 2021). Although the MRI findings in this case were not sufficiently specific to diagnose PNET, they suggested that PNET must be included in the differential list of feline spinal tumours that do not involve external invasion. Furthermore, homogeneous contrast enhancement and a hypointense lesion in T2*WI indicated intratumoural haemorrhage, which may help to facilitate the diagnosis of PNET in cats.

As the reported feline intramedullary tumours are predominantly lymphosarcomas (Marioni‐Henry et al., 2008), few studies have described surgical removal. Long‐term survival was reported in a cat with spinal anaplastic astrocytoma following tumour removal (Tamura et al., 2013). In the present case, progressive myelomalacia was suspected to be the cause of mortality; thus, if this can be avoided, long‐term survival may be possible.

In conclusion, the imaging findings in this report support the inclusion of PNET in the differential diagnosis of tumours confined to the spinal cord in cats.

## AUTHOR CONTRIBUTIONS

*Conceptualization (supporting); data curation (supporting); investigation (supporting); methodology (supporting); visualization (lead); writing – original draft (equal); writing – review and editing (equal)*: Takamasa Itoi, Ikki Mitsui, Natsuki Akashi, Teppei Kanda, Keisuke Sugimoto, Yuki Shimizu and Kazuaki Yamazoe. *Conceptualization (lead); data curation (lead); investigation (lead); methodology (lead); project administration (lead); supervision (lead); visualization (supporting); writing – original draft (equal); writing – review and editing (equal)*: Kenji Kutara.

## CONFLICT OF INTEREST STATEMENT

The authors have no potential conflicts of interest to disclose.

## FUNDING INFORMATION

The authors received no financial support from the research authorship and/or publication of this article.

## ETHICS STATEMENT

This case report was approved by the ethics committee of the Okayama University of Science Veterinary Medical Teaching Hospital (approval number, 2022‐005).

### PEER REVIEW

The peer review history for this article is available at https://publons.com/publon/10.1002/vms3.1283.

## Data Availability

No.
